# Sixth Year of the Symposium on Global Cancer Research: Enhancing Communication and Collaboration to Support Improved Cancer Prevention and Control

**DOI:** 10.1200/JGO.18.00019

**Published:** 2018-03-15

**Authors:** Brenda Kostelecky, Keith Martin, Edward L. Trimble

**Affiliations:** **Brenda Kostelecky** and **Edward L. Trimble**, National Cancer Institute, Rockville, MD; and **Keith Martin**, Consortium of Universities for Global Health, Washington, DC.

March 2018 marks the sixth yearly occasion on which the National Cancer Institute’s (NCI’s) Center for Global Health (CGH) and the Consortium of Universities for Global Health (CUGH) have cohosted the Symposium on Global Cancer Research. The symposium has been held as a satellite meeting of the Annual CUGH Conference to strengthen information exchange about research in the global cancer field and improve cross talk between global cancer professionals and the broader global health community. In this commentary, we outline the rationale behind the NCI-CGH/CUGH partnership in cancer, explore outcomes, and identify next steps as we continue our efforts to address this devastating disease.

The importance of addressing cancer worldwide is underlined by its global burden, with an estimated 8.7 million deaths in 2015, second only to cardiovascular disease.^1^ Contrary to common misperception, cancer is a major health challenge not only in high-income countries but also in low- and middle-income countries (LMICs), where the cancer burden is rapidly growing.^2^ In less-developed regions, chronic infections play an outsized role in causing cancer, where approximately 25% of cancers are estimated to be attributable to infectious agents versus < 10% in highly developed regions.^3^ The burden of cancer in low-income settings is expected to rise substantially with increasing exposure to risk factors in LMICs,^4^ including tobacco use^5^ and obesity.^6^ Resource availability for prevention, screening, and treatment varies greatly worldwide, which creates vast inequities in outcomes.^7^ Complicating matters is that cancer types, causes, and risk factors differ by country and locality.^1,4^ An urgent need to better understand the burden and how to improve prevention, early detection, diagnosis, treatment, and supportive care for populations worldwide underlines the imperative for expanded research and the linking of research to broader health systems improvement efforts.

In recognition of the growing cancer burden and the important role of research in addressing it, NCI-CGH was established in 2011 by then-NCI Director Harold Varmus, MD.^8^ As part of the National Institutes of Health (NIH) within the US Department of Health and Human Services, NCI-CGH was created specifically to coordinate and expand select NCI global cancer research activities and to foster improved incorporation of cancer into the global health agenda. The importance of collaboration with like-minded partners in achieving both objectives was articulated from the outset of NCI-CGH’s development.^8^

CUGH’s establishment in 2008 was supported by generous funding from the Bill & Melinda Gates Foundation, The Rockefeller Foundation, and an initial membership of 24 universities.^9,10^ With recognition that universities play strong roles in education, research, and service that are essential to global improvement in health, CUGH strengthens mutually beneficial and sustained partnerships between academia and other sectors and organizations in diverse countries. CUGH convened its first annual conference at NIH in 2009, where approximately 300 participants attended. The consortium has grown since to include 166 member institutions and many individual members. The 9th Annual CUGH Conference is anticipated to have > 2,000 attendees from > 50 countries.

NCI-CGH and CUGH first began discussions about partnering in a cancer-specific satellite meeting of the Annual CUGH Conference in 2012. At the time, NCI-CGH was conducting an informal landscape analysis of NCI-Designated Cancer Centers’ international activities. It became clear that many cancer centers were working in the same countries, sometimes in the same cities, often with little awareness of the similar initiatives in which other academics were engaged. There were few venues to share timely information about their activities in global cancer research and education. Furthermore, both NCI-CGH and CUGH could see that the scale of the cancer burden in LMICs was greatly underappreciated in the global health community and among the broader public and identified a need to raise awareness of cancer challenges worldwide. CUGH and NCI-CGH viewed a jointly organized satellite meeting to the Annual CUGH Conference as a first step in bringing the academic community and others together to improve communication and collaboration on cancer.

The first Symposium on Global Cancer Research was convened before the 4th Annual CUGH Conference in 2013. The goals of the symposium were threefold: to facilitate information exchange between academics and others working in global cancer, to expand awareness among global health professionals of the cancer burden, and to foster collaboration among groups with shared interests. The symposium format maximized interaction among participants and included roundtables and panel discussions. In addition, CUGH included content on cancer and other noncommunicable diseases (NCDs) in the main conference program. The program included a session on cervical cancer advances relevant to LMICs; a high-level NIH panel that included NCI Deputy Director Douglas Lowy, MD; and remarks by then-Minister of Health of Rwanda Agnes Binagwaho, MD, PhD, in which she highlighted Rwanda’s national human papillomavirus vaccination program to combat cervical cancer.

Positive feedback from participants at the 2013 symposium prompted NCI-CGH and CUGH to continue collaboration and make the Symposium on Global Cancer Research an annual meeting. Subsequent symposia maintained commitment to the original three goals and expanded on the interactive format. A poster session was added to exhibit innovative scientific and implementation-based projects. Travel awards for outstanding abstracts from LMIC participants were granted to bring in fresh perspectives from meritorious investigators abroad. In addition, an award is presented annually to an individual for outstanding scientific and/or humanitarian contributions to global cancer control, which started in 2015.

Local academic partners have been generous in cohosting the symposium to bring innovative ideas, new ways of reaching broader audiences, and a unique local flavor to the satellite meetings. Partner institutions have included the Dana-Farber Cancer Institute in Boston in 2015 and the University of California, San Francisco; the Stanford Cancer Center; and the nonprofit Global Oncology in 2016. In 2018, New York University’s Perlmutter Cancer Center at NYU Langone Health has taken the lead in organizing the symposium in New York City along with cosponsorship from a large contingent of prominent New York region academic centers: Albert Einstein College of Medicine, Herbert Irving Comprehensive Cancer Center at Columbia University, Memorial Sloan Kettering Cancer Center, Tisch Cancer Institute, Ichan School of Medicine at Mount Sinai, Rutgers Global Health Institute, and Rutgers Cancer Institute of New Jersey. In addition, sustained collaboration with ASCO’s *Journal of Global Oncology* to publish symposium abstracts is in its third year (http://ascopubs.org/jgo/meeting-abstracts), which also helps to communicate the outstanding work being done around the world.

Subsequent years have seen an expanded role for highlighting issues in cancer and other NCDs in the Annual CUGH Conference. A program track focused on NCDs was added to highlight original projects and programs in special sessions, oral presentations, and posters. Several prominent speakers have been featured in panel and plenary presentations, including NIH Director Francis Collins, MD, PhD; former NCI Director Varmus; former National Heart, Lung, and Blood Institute Acting Director Susan Shurin, MD; Fogarty International Center Director Roger Glass, MD; former Centers for Disease Control and Prevention Director Tom Frieden, MD; Vice President of Global Cancer Control, American Cancer Society, Ambassador Sally Cowal; South African Medical Research Council President Glenda Gray, MBBCH; and US President Barack Obama, who sent a video message of his ardent support for the community's collective work in global health. Many sessions in the past several years have brought together key leaders on important topics, such as integration of tobacco control into infectious disease programs, cancer prevention and control as part of women’s health programs, and NCDs in low-income countries. The inclusion of such speakers and sessions has improved the visibility of global cancer and helps to drive discussions and debate on how NCDs can be addressed in global health.

The symposium has achieved many of its aims and is an important regular meeting point to exchange knowledge on recent scientific progress and novel program ideas. Participant surveys after the 2014 to 2017 symposia demonstrated that the program components were relevant to > 90% of participants. Similarly high percentages of participants believed the program to be useful for their work. Respondents to the questionnaires consistently commented on the usefulness of the interactive sessions and especially the poster sessions for networking, keeping abreast of the field, and identifying potential collaborators. Continued robust and provocative discussion at this and other conferences can help to catalyze additional collaborations as well as continued training and education initiatives. NCI-CGH currently is collaborating with ASCO to conduct a new landscape analysis of the NCI-Designated Cancer Centers’ international work. This systematic analysis will help to identify gaps to address through future symposia and/or broader collaborative initiatives with partners such as CUGH.

The 2018 Annual CUGH Conference theme is Health Disparities: A Time for Action (www.cugh2018.org) and is aligned with the symposium’s theme Addressing Disparities, Locally and Globally. Together, the meetings will have many sessions relevant to the NCI-CGH/CUGH partnership goals, including the future of cancer health disparities research, new technology for cancer control, cancer early detection, lessons from HIV, public-private partnerships, improvement of access to surgical care and medicines, cervical cancer screening, NCD services among refugees, social determinants of health, palliative care, and improvement of data quality. In addition, the conference will feature the release of the Lancet Commission on Pathology, a Lancet workshop on academic writing, a Pulitzer workshop on communication, and a session that highlights the African Forum for Research and Education in Health. The conference’s Global Health Film Festival will exhibit several short, powerful documentaries on global health challenges.

Over the 6 years of our collaboration, we at NCI-CGH and CUGH have been pleased to see growing recognition of cancer and NCDs as important components of global health. The conference and symposium have provided an exceptional environment where academics and representatives from various US government departments and agencies involved in global health (NIH, Centers for Disease Control and Prevention, Department of Health and Human Services, US Agency for International Development, Environmental Protection Agency, and more) can share knowledge and network with one another and the broader global health community. Progress is needed to bring professionals together from various health areas as well as to integrate non–health sectors in bringing about effective solutions that address the social determinants of health. The translation of known, evidence-based interventions into policy remains a great challenge of our time but one in which the scientific community is uniquely placed to lead. Ultimately, cohesive and sustained collaboration between committed partners toward mutually beneficial solutions will be needed to create an enduring reduction in cancer rates and an improvement in care. With lessons learned from the past 6 years, we aim to move forward to strengthen global cancer research to find viable solutions to this high-burden disease and to incorporate this important health problem into the global health agenda.

## References

[1] Fitzmaurice C Allen C Barber RM et al Global, regional, and national cancer incidence, mortality, years of life lost, years lived with disability, and disability-adjusted life-years for 32 cancer groups, 1990 to 2015: A systematic analysis for the Global Burden of Disease Study JAMA Oncol 3 524 548 2017 27918777 10.1001/jamaoncol.2016.5688 PMC6103527

[2] Vineis P Wild CP Global cancer patterns: Causes and prevention Lancet 383 549 557 2014 24351322 10.1016/S0140-6736(13)62224-2

[3] Plummer M de Martel C Vignat J et al Global burden of cancers attributable to infections in 2012: A synthetic analysis Lancet Glob Health 4 e609 e616 2016 27470177 10.1016/S2214-109X(16)30143-7

[4] Bray F Soerjomataram I The changing global burden of cancer: Transitions in human development and implications for cancer prevention and control Gelband H Jha P Sankaranarayanan R et al Cancer: Disease Control Priorities, Volume 3 ed 3 Washington, DC International Bank for Reconstruction and Development/The World Bank 2015 26913347

[5] Giovino GA Mirza SA Samet JM et al Tobacco use in 3 billion individuals from 16 countries: An analysis of nationally representative cross-sectional household surveys Lancet 380 668 679 2012 22901888 10.1016/S0140-6736(12)61085-X

[6] Tanumihardjo SA Anderson C Kaufer-Horwitz M et al Poverty, obesity, and malnutrition: An international perspective recognizing the paradox J Am Diet Assoc 107 1966 1972 2007 17964317 10.1016/j.jada.2007.08.007

[7] Horton S Gauvreau CL Cancer in low- and middle-income countries: An economic overview Gelband H Jha P Sankaranarayanan R et al Cancer: Disease Control Priorities, Volume 3 ed 3 Washington, DC International Bank for Reconstruction and Development/The World Bank, 2015 26913333

[8] Varmus H Trimble EL Integrating cancer control into global health Sci Transl Med 3 101cm28 2011 10.1126/scitranslmed.3002321 21937755

[9] Martin K Global challenges, global opportunities Ann Glob Health 80 153 154 2014 25257540 10.1016/j.aogh.2014.06.001

[10] Consortium of Universities for Global Health http://www.cugh.org

